# Pneumoperitoneum in the Newborn: Is Surgical Intervention Always Indicated?

**Published:** 2014-07-10

**Authors:** Rahul Gupta, Shyam Bihari Sharma, Priyanka Golash, Ritesh Yadav, Dhawal Gandhi

**Affiliations:** 1 Department of Pediatric Surgery, Nims University, Shobha Nagar, Jaipur-303121, Rajasthan; 2 Department of Pediatrics, Nims University, Shobha Nagar, Jaipur-303121, Rajasthan

**Keywords:** Newborn, Spontaneous, Pneumoperitoneum, Conservative management

## Abstract

Pneumoperitoneum in the neonate generally is an acute surgical emergency, which has grave implications, and immediate surgical intervention is needed to ensure survival. The most common cause is a perforated hollow viscus. However, there are causes that cannot be attributed to this etiology, constituting what has been called non-surgical, asymptomatic, benign, misleading, spontaneous or idiopathic pneumoperitoneum. Knowledge of this entity and its likely aetiological factors should improve awareness and possibly reduce the imperative to perform an unnecessary emergency laparotomy on an otherwise normal neonate with an unexplained pneumoperitoneum.

## INTRODUCTION

 Pneumoperitoneum in the neonate, generally is an acute surgical emergency which has grave implications, and immediate surgical intervention is needed to ensure survival, but it may not always be an absolute indication for surgery in neonate.[1,2] Spontaneous Pneumoperitoneum in a newborn without peritonitis, with a normal abdominal examination, is an extremely rare event which creates a dilemma for the treating surgeon regarding surgical management of the neonate. Conservative management, avoiding a laparotomy is the treatment of choice. We present herein a case of spontaneous pneumoperitoneum in a newborn who was successfully treated at our institute. 

## CASE REPORT

A36-week 2.1-kg preterm, female baby born to a primigravida mother by normal vaginal delivery, presented to us within first few hours of birth with respiratory distress. The mother received adequate antenatal and perinatal care and there was no history of birth asphyxia. On examination, the baby was alert but having respiratory distress in the form of grunting and subcostal recession, pulse rate-180/min, SO2-85%. The patient was stabilized and resuscitated with oropharangeal suctioning, nebulisation, oxygen therapy, intravenous fluids, antibiotics and a nasogastric tube was placed to decompress the stomach. Continuous Positive Airway Pressure (CPAP) support was given. The condition of the neonate improved. On the next day, although the general condition of the baby was stable, she developed abdominal distension and non-passage of the meconium. Abdomen was moderately distended, however, there was no erythema, tenderness or, a palpable lump [Fig.1a].The laboratory investigations were within normal range. Abdominal and chest radiographs revealed pneumoperitoneum, with free gas under both the domes of diaphragm [Fig.2a]. There was no evidence of pneumothorax or pneumomediastinum, bilateral lung fields were clear. Ultrasound showed normal domes of diaphragm.

In view of the stable general condition and without clinical evidence of necrotizing enterocolitis (NEC), pneumothorax, pneumomediastinum, and also absence of preceding event which could lead to gastrointestinal perforations, the diagnosis of spontaneous pneumoperitoneum was made. It was decided to continue with the conservative treatment. The patient was kept under close monitoring with frequent abdominal girth measurements to detect any deterioration in the condition. The abdominal distension resolved [Fig.1b]. The baby passed meconium plugs initially followed by normal meconium on administration of saline enema. Repeat abdominal radiographs showed resolution of pneumoperitoneum [Fig.2b]. A trial feed was well tolerated and was slowly graduated to full feeds by the seventh day of admission. The neonate was discharged and is doing well on follow-up.

**Figure:**
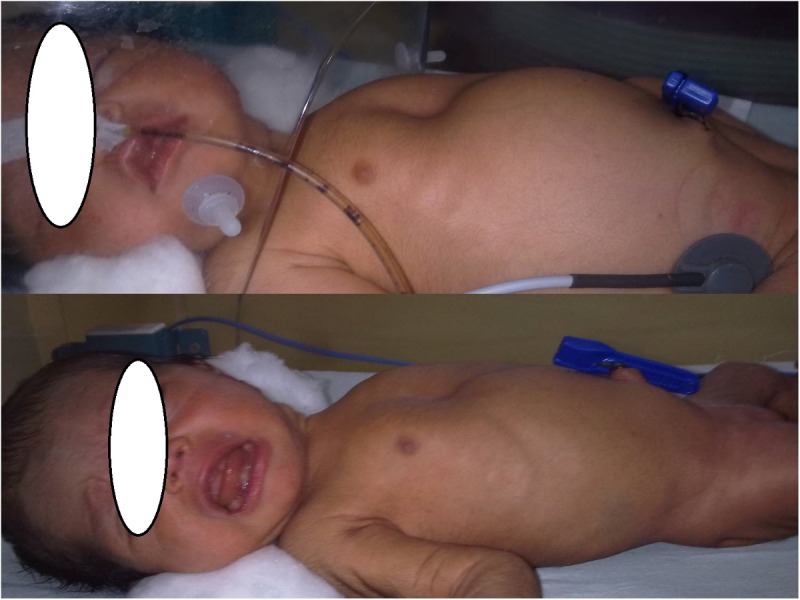
Figure 1: (a) Clinical photographs of the neonate with abdominal distension (top). (b) Clinical photograph after resolution of spontaneous Pneumoperitoneum (bottom).

**Figure:**
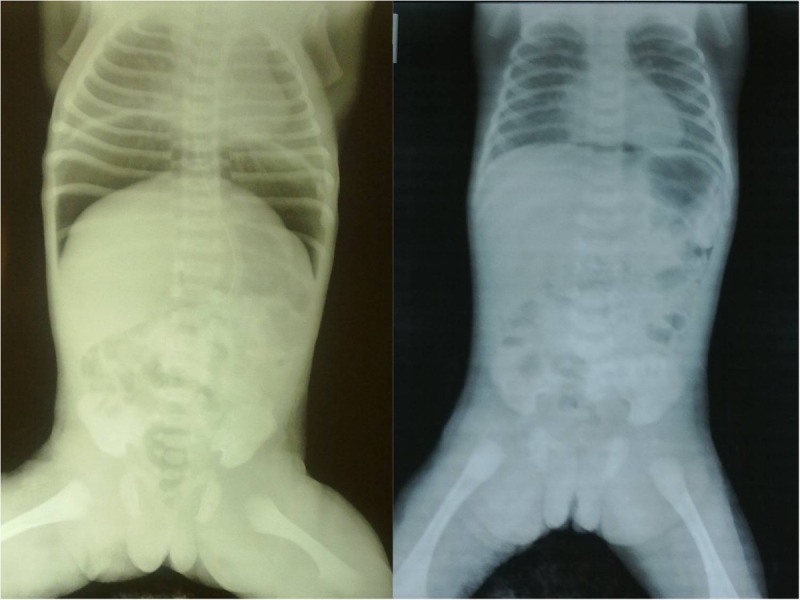
Figure 2: (a) Radiographs revealing the pneumoperitoneum (left). (b) Radiographs revealing resolution of spontaneous pneumoperitoneum after conservative management (right).

## DISCUSSION

Besides NEC which is the most common cause of pneumoperitoneum in a neonate, there are numerous other surgical causes of pneumoperitoneum and these include gastric and duodenal perforations, isolated colonic perforations, caecal perforations, perforated pouch colon, idiopathic gastric necrosis, and necrosis of small intestine or colon secondary to intestinal atresia, volvulus, meconium ileus, Hirschsprung's disease or Meckel's diverticulum.[3] Other rare causes of neonatal intestinal perforation could be mechanical injury from the gavage tubes, rectal thermometers, rectal tubes used for rectal washes, resuscitation with oxygen under pressure in patients with distal pyloric or duodenal obstruction, congenital defects of the musculature and diverticula.[3,4] The usual accepted mode of treatment of neonates with pneumoperitoneum and necrotizing enterocolitis (NEC) with pneumoperitoneum is operative management.

Since 1966, when an article had concluded that every newborn infant with pneumoperitoneum must undergo laparotomy, a lot of paradigm shift in the management has occurred over the over last few decades.[5] It is the result of increasing awareness regarding the entity called spontaneous pneumoperitoneum, among experienced paediatric surgeons. It is also known as non-surgical, asymptomatic, benign, misleading, spontaneous or idiopathic pneumoperitoneum.[1,6,7]

As spontaneous pneumoperitoneum has been earlier reported in premature infants who are on mechanical ventilation for pulmonary or cardiac diseases.[1,8] In the index case, all of the earlier mentioned causes were absent and pneumoperitoneum was the result of oxygen therapy in the form of CPAP. The pathophysiology of the appearance of pneumoperitoneum could possibly be extension of free air under tension in the mediastinum along the vascular planes through the normal diaphragmatic openings, retrograde path through the pulmonary lymphatics, congenital diaphragmatic hernia or from a pleuroperitoneal fistula.[1,2,6-10] 

A flow chart [Fig. 3] to explain the mechanism in the development of spontaneous pneumoperitoneum is proposed after imbibing the ideas from the works of Macklin and Williams.[1,10]

**Figure:**
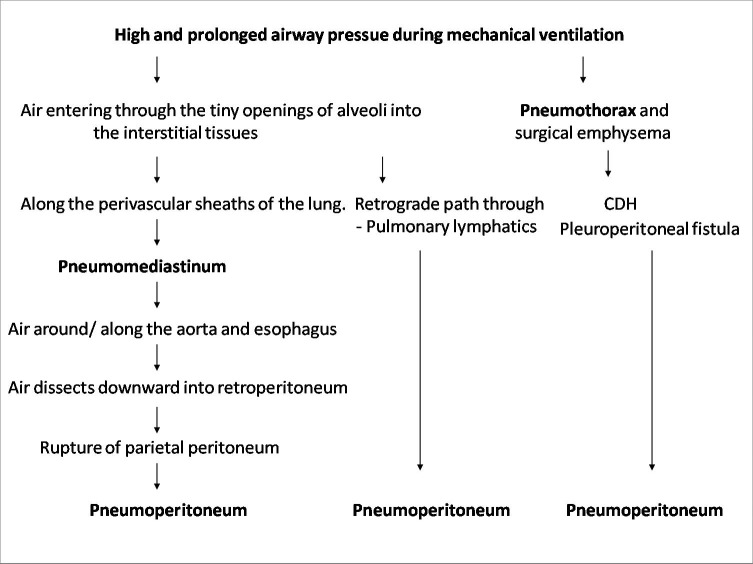
Figure 3: A flow chart to explain the mechanism in the development of spontaneous pneumoperitoneum.

Presence of pneumothorax or pneumomediastinum immediately before or simultaneously with pneumoperitoneum is a pointer towards spontaneous pneumoperitoneum, although it may be absent in few cases, which happened in our case.[6-10]

A neonate with radiographs showing pneumoperitoneum requires a thorough clinical (abdominal and pulmonary signs) and laboratory correlation (blood parameters and blood gases) to establish the aetiology of perforation to direct further management. The indications for surgical intervention in pneumoperitoneum are the features of peritonitis, pain, cardiovascular instability, leucocytosis, evidence of leakage from gastrointestinal tract and failure of conservative management.[1]

## Footnotes

**Source of Support:** Nil

**Conflict of Interest:** None

